# Hydrogen-bond-mediated structural variation of metal guanidinium formate hybrid perovskites under pressure

**DOI:** 10.1098/rsta.2018.0227

**Published:** 2019-05-27

**Authors:** Zhengqiang Yang, Guanqun Cai, Craig L. Bull, Matthew G. Tucker, Martin T. Dove, Alexandra Friedrich, Anthony E. Phillips

**Affiliations:** 1 School of Physics and Astronomy, Queen Mary University of London, London E1 4NS, UK; 2 ISIS Neutron and Muon Source, Rutherford Appleton Laboratory, Chilton, Didcot, Oxon OX11 0QX, UK; 3 Institut für Geowissenschaften, Goethe-Universität Frankfurt, Altenhöferallee 1, Frankfurt am Main 60438, Germany

**Keywords:** hybrid perovskites, coordination frameworks, phase transitions, high pressure, hydrogenbonding

## Abstract

The hybrid perovskites are coordination frameworks with the same topology as the inorganic perovskites, but with properties driven by different chemistry, including host-framework hydrogen bonding. Like the inorganic perovskites, these materials exhibit many different phases, including structures with potentially exploitable functionality. However, their phase transformations under pressure are more complex and less well understood. We have studied the structures of manganese and cobalt guanidinium formate under pressure using single-crystal X-ray and powder neutron diffraction. Under pressure, these materials transform to a rhombohedral phase isostructural to cadmium guanidinium formate. This transformation accommodates the reduced cell volume while preserving the perovskite topology of the framework. Using density-functional theory calculations, we show that this behaviour is a consequence of the hydrogen-bonded network of guanidinium ions, which act as struts protecting the metal formate framework against compression within their plane. Our results demonstrate more generally that identifying suitable host–guest hydrogen-bonding geometries may provide a route to engineering hybrid perovskite phases with desirable crystal structures.

This article is part of the theme issue ‘Mineralomimesis: natural and synthetic frameworks in science and technology’.

## 1. Introduction

The hybrid perovskites are a family of materials analogous in structure to the inorganic perovskites. In both the inorganic and hybrid materials, ‘**B**-site’ cations are linked by anions into a cubic network, with ‘**A**-site’ cations occupying the cubic interstices. In the hybrid materials, however, a relatively large linker anion such as iodide, cyanide or formate expands the network compared to the inorganic analogues, allowing the interstitial **A** site to be occupied by a polyatomic organic ion such as an alkylammonium, guanidinium, formamidinium, acetamidinium or imidazolium. Like their inorganic analogues, the hybrid materials exhibit both a great diversity of potential compositions, with hundreds of these materials reported over the past decade [1,2], and important functionality, most famously including solar energy conversion [3] but also ferroelectric [4] and caloric behaviour [5].

There is every reason to expect the phase diagrams of this family of materials to be as rich as those of their inorganic counterparts [6]. Indeed, because the polyatomic linker anions lend the frameworks greater flexibility, we might anticipate an even greater diversity of phases in the hybrid materials. This phase transition behaviour will depend on fundamentally new physics and chemistry. In contrast with the inorganic perovskites, organic **A**-site cations have a shape—more formally, they may have intrinsic electric dipole or higher-order multipole moments [7]—which will strongly influence their structure and properties. Similarly, hydrogen bonding between the organic guest cation and anionic framework may dramatically change the relative stability of different structures. These effects have only recently begun to be explored and remain poorly understood. Yet mapping and understanding phase transitions in the hybrid perovskites is both of intrinsic interest from a crystal engineering perspective and of great value for potential applications, as a means to tune these materials' electrical and magnetic properties.

The best-explored variable in the phase diagrams of the hybrid perovskites is temperature, with many phase changes with respect to temperature now known [4]. On the other hand, with the exception of the well-studied lead halide perovskite semiconductors [8–10], relatively few structural studies of materials in this family under applied pressure have been reported [11–13], although in some cases vibrational spectroscopy has intriguingly indicated structural changes [14,15]. In particular, spectroscopic methods have revealed high-pressure changes in many metal formate hybrid perovskites [16–21].

One common trend in hybrid perovskites with disordered **A**-site guests is that the decrease in void space with pressure causes these cations to freeze into an ordered configuration; this has now been established in both halide [22,23] and formate perovskites [24,25]. Here, we consider instead a family of materials, the metal guanidinium formates, in which the **A**-site guests are ordered under ambient conditions. We report single-crystal synchrotron X-ray diffraction, powder neutron diffraction and density-functional theory (DFT) calculations on these compounds under pressure. Our results demonstrate that the phase diagrams of these materials are dictated by hydrogen-bonding interactions between the guanidinium and formate ions, with implications for crystal engineering of the hybrid perovskites more generally.

## 2. Target materials

**Figure 1.**
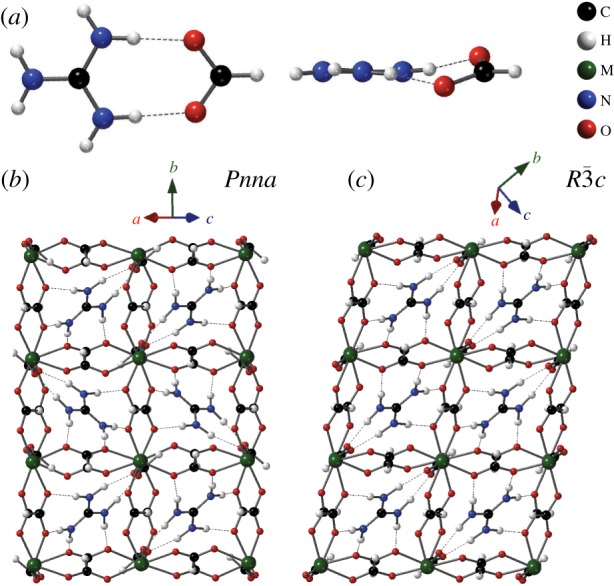
(*a*) In the crystal structures discussed here, the guanidinium (N(CH_2_)^+^_3_) and formate (HCO^−^_2_) ions have a snug hydrogen-bonded fit. Depending on the metal ion, two metal guanidinium formate perovskite structures are known at ambient pressure. (*b*) An orthorhombic phase in which guanidinium ions lie in two differently oriented planes, alternating down each column. (*c*) A rhombohedral phase (referred here to hexagonal axes) in which all guanidinium ions lie in parallel planes. (Online version in colour.)

In the metal guanidinium formates, C(NH_2_)_3_[**M**^II^(HCO_2_)_3_] (henceforth **MGF**), the metal ions **M** are linked by formate ions into a network, with the guanidinium ions occupying the cubic interstices [26]. The guanidinium ions act as struts that support the framework through the snug hydrogen-bonded fit between guanidinium and formate ions (figure 1*a*). As a result of this strong interaction, the guanidinium ions are crystallographically ordered, in contrast with, for instance, the dimethylammonium metal formates [27] and the guanidinium metal cyanides [28], where the guest–framework interaction is weaker and the guest ions are disordered at room temperature.

The materials in this family with **M** = Mn, Fe, Co, Ni, Zn have an orthorhombic structure (space group *Pnna*), in which the pseudocubic perovskite cell is distorted slightly along the face diagonal. In this structure, alternate planes of guanidinium ions are tilted in different directions, forming a herringbone pattern (figure 1*b*). By contrast, **CdGF** adopts a rhombohedral structure (space group $R\bar{3}c$) in which the pseudocubic cell is distorted along the body diagonal [29]. In this form, each guanidinium ion lies in a parallel plane (figure 1*c*).

We have studied the behaviour of **MnGF** and **CoGF** under pressure, using single-crystal laboratory and synchrotron X-ray and powder neutron diffraction. We found that both **MnGF** and **CoGF** undergo a first-order transition from the ambient orthorhombic phase to a rhombohedral phase isostructural with **CdGF** at moderate pressures, with the two phases in each case coexisting over a small pressure range (**MnGF**, 1.2 GPa to 1.5 GPa; **CoGF**, 2.0 GPa to 2.6 GPa). Unlike the related metal ammonium formates in argon pressure-transmitting medium, no indication of the medium entering the framework was observed [11]. In single-crystal measurements, the high-pressure phase exists as a non-merohedral twin, with two components corresponding to the two orientations of the guanidinium ions in the ambient-pressure herringbone pattern (see electronic supplementary material). Indeed, parallel twin domains are clearly visible in the high-pressure phase (electronic supplementary material, figure S1). Taking layers of the two different guanidinium orientations to represent ‘spin up’ and ‘spin down’, the system is thus analogous to a one-dimensional Ising spin chain: initially antiferromagnetic, applying pressure causes the nearest-neighbour interactions to become ferromagnetic, and hence domains of aligned guanidinium ions grow to macroscopic sizes.

Here, we will first discuss the behaviour within each phase and then consider the reasons for the phase transition itself.

## 3. Strain

**Figure 2.**
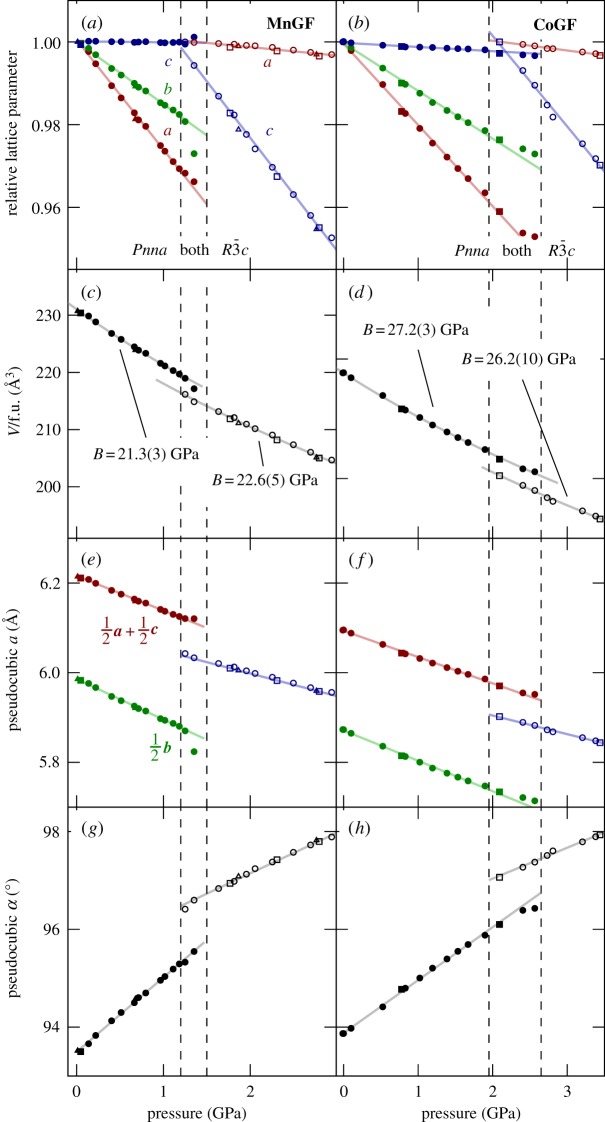
Crystallographic unit-cell parameters of (*a*) **MnGF** and (*b*) **CoGF**, relative to the lowest-pressure values in each phase, as determined from powder neutron diffraction. Normalized crystallographic unit-cell volumes (per formula unit) along with fitted bulk moduli (see electronic supplementary material) are shown in (*c*,*d*). The same data can be alternatively visualized in terms of the pseudocubic perovskite (*e*,*f* ) cell length *a* and (*g*,*h*) lattice angle *α*. Closed symbols represent the orthorhombic phase, open symbols the rhombohedral one; different symbols (circle, triangle, square) correspond to different sample loadings. (Online version in colour.)

It is instructive to examine the structural variation within each phase in two different ways. First, we can simply plot the relative change of each lattice parameter on applying pressure (figure 2*a*,*b*). In both the orthorhombic and the rhombohedral phases, the linear compressibility varies substantially between the crystallographic axes (table 1). At the most extreme example, in the orthorhombic phase of **MnGF**, the linear compressibility along the *a*-axis is substantial while that along the *c*-axis is within experimental error of zero. This behaviour is readily understandable in terms of the orientation of the guanidinium ions. These ions act as struts, keeping the framework relatively rigid within their plane (figure 1) while preserving void space above and below this plane, allowing compression in the perpendicular direction. In the orthorhombic phase, the *c*-axis runs parallel to the plane of every guanidinium ion, while the *a*- and *b*-axes are angled away from these planes; thus the linear compressibility is far greater along the *a*- or *b*-axes than along *c*. By contrast, in the rhombohedral phase, the guanidinium ions lie in the *ab* plane, and the linear compressibility is hence greater along *c* than along *a* or *b*. The net effect in both materials is that the two phases have comparable bulk moduli (figure 2*c*,*d*).

**Table 1.** Linear compressibilities −∂ℓ/ℓ∂*P* (TPa^−1^) of the target materials in the orthorhombic and rhombohedral phases, estimated from straight-line fits to the crystallographic data shown in figure 2.

**axis**	**MnGF**	**CoGF**
orthorhombic		
*a*	26.7(4)	19.5(3)
*b*	15.2(2)	11.4(2)
*c*	0.03(16)	1.10(8)
rhombohedral (hexagonal axes)		
*a* = *b*	2.04(15)	2.34(10)
*c*	27.3(5)	21.9(7)

A second way to examine these data is to transform the lattice parameters to a pseudocubic cell corresponding to the cubic perovskite aristotype. In the orthorhombic phase, this pseudocubic cell has two independent cell lengths and one variable angle (with the other two fixed at 90°); in the rhombohedral phase, the pseudocubic cell's three lengths and three angles are, respectively, identical. Analysing the data in this fashion shows by contrast that the pseudocubic cell lengths *a* decrease in both phases of both materials at an approximately constant rate (figure 2*e*,*f* ), while the pseudocubic cell angle *α* increases (figure 2*g*,*h*). In each case, this reflects the compression and collapse that would be expected of a topologically cubic framework under pressure.

Of course, these two analyses contain exactly the same information, but they highlight different aspects. While applying pressure causes the cubic metal formate framework to collapse, the guest guanidinium ions act as relatively incompressible struts.

## 4. Phase transition

We now turn to the phase transition itself. It is familiar behaviour that, starting from the orthorhombic phase of **MnGF** or **CoGF**, the same rhombohedral phase can be achieved either by applying pressure or by increasing the cation size. For instance, many NaCl-type alkali halides and pseudohalides transform to the CsCl structure under pressure [30]. Considering the inorganic perovskites, in a similar way the ‘post-perovskite’ phase of MgSiO_3_ adopts the CaIrO_3_ structure [31]. These phase transitions each involve a change in coordination number about the metal ions, and hence a change in the bonding topology.

It is more common in the inorganic perovskites for applied pressure to induce distortions that preserve the cubic topology, often in a similar sequence to that induced by temperature. In these cases, as predicted by the Goldschmidt tolerance factor formalism, a similar relationship may hold between transitions induced by pressure and by cation size. For instance, BaTiO_3_ (*r*_Ti_ = 0.605 Å) has a tetragonal structure, with **B**-site Ti cations displaced from the centre of their octahedra, at ambient temperature and pressure; this transforms under pressure to the cubic perovskite aristotype. Increasing the size of the **B**-site cation, BaSnO_3_ (*r*_Sn_ = 0.69 Å) has the cubic structure under ambient conditions [32]. However, such phase transitions between states that are very similar in free energy are more subtle, and there are also many instances in which this simple heuristic does not hold.

In the metal formate perovskites, all known phase transitions are of this second type, preserving the cubic network topology. Indeed, this topology is even recoverable from a pressure-induced amorphous phase [25]. Certainly, this is the case for the **MGF** compounds: the two phases have the same coordination number and topology, but differ instead in the orientation of the **A**-site guanidinium cations. To our knowledge, this is the first reported phase transition between two *ordered* phases of a hybrid perovskite with different guest ion orientations.

**Figure 3.**
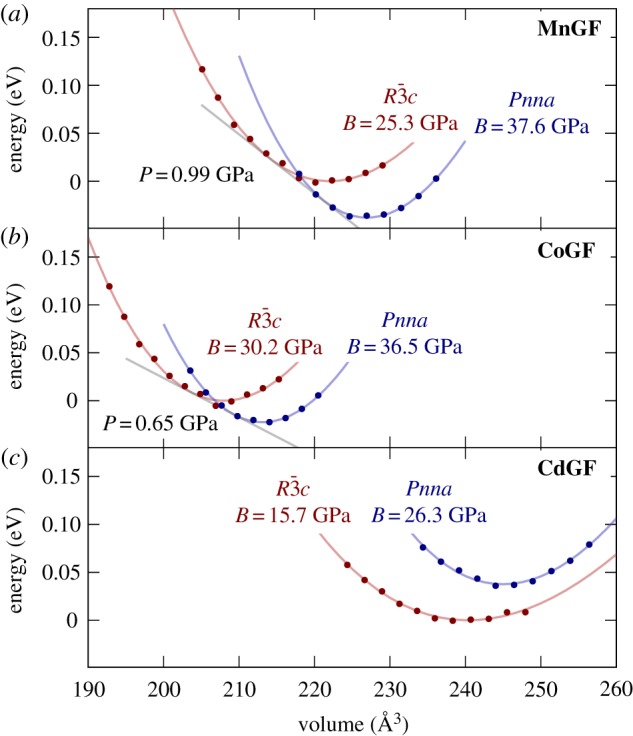
Lattice energy as a function of volume of the rhombohedral and orthorhombic phases from DFT geometry optimizations at constant volume, with fits to the second-order Birch–Murnaghan equation of state, for (*a*) **MnGF**, (*b*) **CoGF** and (*c*) **CdGF**. Both energy and volume are given per formula unit. The fitted bulk modulus *B* is labelled on the graph; full fitted parameters are given as electronic supplementary material. (Online version in colour.)

To elucidate this behaviour, we used DFT calculations to determine the energy of each phase as a function of volume for **MnGF**, **CoGF** and **CdGF**. In agreement with the experimental data, our results show that in **MnGF** and **CoGF**, the orthorhombic phase is the most stable at zero pressure, while the rhombohedral phase, with smaller volume, is favoured at higher pressures (figure 3*a*,*b*). The predicted transition pressures are 0.99 GPa (**MnGF**) and 0.65 GPa (**CoGF**). It is not surprising that these values are both smaller than those observed experimentally: this first-order phase transition involves substantial rearrangement of the guanidinium ions, and the pressure at which the two phases nominally have the same enthalpy should thus be considered a lower bound rather than a quantitative prediction of the phase transition pressure. Indeed, one might expect that in the larger Mn cell this rearrangement should be slightly easier than in the smaller Co analogue, rationalizing the observations that both (1) the difference between the nominal DFT and experimentally observed phase transition pressures and (2) the pressure range where the phases coexist are smaller for **MnGF** than for **CoGF**. In particular, we suggest that such kinetic effects may explain why the phase transition is experimentally observed at a higher pressure for **CoGF** than for **MnGF**, while the DFT model predicts the reverse. Alternatively, of course, this discrepancy may simply reflect limitations of the modelling approach.

By contrast, in **CdGF**, although the most stable orthorhombic structure again has a greater volume than the most stable rhombohedral structure, the rhombohedral phase is favoured at all cell volumes (figure 3*c*). Again, this is in agreement with the experimental observation that no orthorhombic phase has been observed in this material.

## 5. Discussion

The difference between these materials' behaviour can be rationalized in terms of the hydrogen bonding between guanidinium and formate ions. Table 2 shows the N–H · *s*O distance in **MnGF** and **CdGF**, as determined from single-crystal X-ray diffraction experiments and DFT calculations. In each case, the DFT values are 0.05 Å to 0.08 Å smaller than the diffraction results at ambient temperature and pressure. In **MnGF**, the DFT N · *s*O distances fall by at most 0.025 Å across the phase transition. Even considering the energy minima, rather than the structures immediately before and after the phase transition, the differences in distance between the phases range from 0.03 Å to 0.04 Å. This result is slightly smaller than the experimental values of 0.03 Å to 0.09 Å, where, to provide the most accurate experimental comparison, we have combined the atomic coordinates from single-crystal diffraction with lattice parameters from powder diffraction at a pressure where the phases coexist.

**Table 2.** N–H · *s*O distances, in the orthorhombic and rhombohedral phases of **MnGF** and **CdGF**, from single-crystal X-ray diffraction (SCXRD), powder neutron diffraction (PND) and DFT modelling. For the powder neutron data, the atomic coordinates were fixed at their value from single-crystal X-ray models at the nearest available pressure.

**material**	**conditions**	**phase**	**N–**H · *s*O distance (Å)
**MnGF**	SCXRD, 0 GPa, 293 K [26]	*Pnna*	2.9529(19), 2.976(2), 2.9904(16)
	PND, 1.25 GPa, ambient *T*	*Pnna*	2.906(5), 2.919(5), 2.965(5)
		$R\bar{3}c$	2.879(10)
	DFT, minimum energy	*Pnna*	2.8964, 2.8968, 2.9136
	DFT, minimum energy	$R\bar{3}c$	2.8705
	DFT, 220 Å^3^	*Pnna*	2.8580, 2.8639, 2.8749
	DFT, 214 Å^3^	$R\bar{3}c$	2.8498
**CdGF**	DFT, minimum energy	*Pnna*	2.9883, 2.9883, 2.9883
	DFT, minimum energy	$R\bar{3}c$	2.8736
	SCXRD, 0 GPa, 300 K [29]	$R\bar{3}c$	2.927(3)

On the other hand, in **CdGF**, the difference in the DFT N · *s*O distance between the two energy minima is 0.11 Å, four times the corresponding value in **MnGF**. Moreover, the absolute DFT N · *s*O distance in the putative orthorhombic phase, 2.99 Å, is substantially larger than the DFT value from any experimentally observed phase (2.87 Å to 2.91 Å). Thus it seems that the hypothetical orthorhombic unit cell in **CdGF** is both too large and too rigid to allow effective hydrogen bonding.

This is consistent with our observations of these structures' flexibility more generally. As previously noted, the rhombohedral structure is distorted along the pseudocubic body diagonal (i.e. the hexagonal *c*-axis; figure 1*c*), which is perpendicular to all guanidinium ions, and is therefore relatively flexible along this direction. On the other hand, the herringbone arrangement of guanidinium ions makes the orthorhombic structure more rigid. Thus the rhombohedral structure is able to accommodate favourable hydrogen-bonding distances in both **MnGF** and **CdGF**; by contrast, the more rigid orthorhombic structure is unable to distort in this way.

At this point, we pause to consider the extent and nature of the agreement between our experimental and computational data. In addition to uncertainties associated, for instance, with the specific choice of exchange–correlation functional, the DFT methodology used here has two features that fundamentally differentiate it from experiment. First, DFT does not take thermal vibrations into account, and thus effectively simulates a classical crystal at absolute zero temperature. Second, fitting optimized energy as a function of cell volume considers only distortions at the gamma point (that is, those in which every unit cell distorts in the same way).

These differences allow us to account for several apparent discrepancies between the experimental and computational results. First, the DFT underestimates the unit-cell volume and hence overestimates the bulk modulus. This is a natural consequence of neglecting thermal expansion. Second, the DFT predicts that the orthorhombic phase is mechanically stiffer than the rhombohedral one (figure 3), while the experimental bulk moduli of the phases are similar (figure 2*c*,*d*). Again, this can be explained in terms of the points above. Considering only distortions at the gamma point and zero temperature, the modelling shows that compression of the rhombohedral phase is easier than for the orthorhombic structure, presumably because of the rhombohedral phase's flexibility along the hexagonal *c*-axis, discussed previously. On the other hand, the experimental results will reflect the influence of thermally excited vibrational modes, including those at other wavevectors. Importantly, the DFT isolates the specific sense of flexibility that we argue is responsible for the phase transformation behaviour: the ability of the rhombohedral metal formate framework to accommodate the guanidinium ions at a variety of unit-cell volumes. Thus the difference between experimental and simulated bulk moduli does not contradict our argument above.

As a final comparison, we consider related manganese(II) formate perovskites in which host–guest hydrogen bonding is less important. In dimethylammonium manganese formate, under ambient conditions, the manganese formate framework has the same rhombohedral structure as discussed above, with the dimethylammonium ions disordered about the threefold axis for want of a strongly bound hydrogen-bonding site [27,33]. This suggests that host–guest hydrogen bonding is not needed to stabilize the rhombohedral phase. An even more dramatic example is provided by the material ‘[Mn(HCO_2_)_3_] · *n*H_2_O’, which has no bulky **A**-site cation at all. Under ambient conditions, it has the same rhombohedral structure as discussed above, with guest water molecules occupying the cubic interstices (*a* = 8.327 Å, *c* = 22.890 Å) [34]. The original report suggested that this compound contains manganese(III) ions. However, the crystals were colourless and the Mn–O bond lengths were 2.190 Å; both of these observations suggest that the correct oxidation state is manganese(II) [35], with charge balance preserved by a guest hydronium ion, [Mn(HCO_2_)_3_] · H_3_O · *n*H_2_O. (For comparison, the closely related compound $[\mathrm{Mn}{\left({\mathrm{HCO}}_{2}\right)}_{3}]\cdot \frac{1}{2}{\mathrm{CO}}_{2}\cdot \frac{1}{4}\mathrm{HCOOH}\cdot \frac{2}{3}H_{2}O$, which unambiguously contains manganese(III) ions, is dark red and has an Mn–O bond length of 2.001 Å [36].) If this oxidation state assignment is accepted, then this material demonstrates that the rhombohedral structure is stable even in the absence of a bulky organic **A**-site cation.

## 6. Conclusion

In conclusion, we have identified a new high-pressure phase in the guanidinium metal formate perovskites **MnGF** and **CoGF** that is isostructural with the ambient-pressure structure of **CdGF**. Our experimental and modelling data demonstrate that the host–guest hydrogen bonding between guanidinium and formate ions plays a crucial role in determining which phase is the more stable: the rhombohedral structure is able to accommodate both small (**MnGF** and **CoGF** under pressure) and large (**CdGF**) unit cells, while the orthorhombic structure provides a snug fit for **MnGF** and **CoGF** at ambient pressure but cannot easily distort to accommodate the larger Cd ion. Host–guest hydrogen-bonding interactions also strongly influence distortion within each phase, with the linear compressibility being notably smaller in directions where the guanidinium ions are able to resist compression by acting as ‘struts’ within the framework.

More generally, our results provide a further demonstration of the complex interplay between framework and guest in determining the structures of the hybrid perovskites. In contrast with their inorganic analogues, host–guest hydrogen bonding may stabilize particular structures but only over a relatively small pressure, temperature or composition range. The complexity of the resulting phase diagrams invites substantial further investigation of the consequences for these materials' properties.

## Supplementary Material

Laboratory data and models: MnGF

## Supplementary Material

Synchrotron data and models: MnGF

## Supplementary Material

Synchrotron data and models: CoGF

## Supplementary Material

Experimental and computational details

## Data accessibility

The raw neutron data reported here are available from the ISIS data repository at DOIs 10.5286/ISIS.E.47628012 and 10.5286/ISIS.E.58447525. Single-crystal diffraction data in CIF format and full experimental and computational details are provided as electronic supplementary material.
