# Supplementary material for: Hydrogen-bond-mediated structural variation of metal guanidinium formate hybrid perovskites under pressure
Source: Philos Trans A Math Phys Eng Sci. 2019 May 27;377(2149):20180227. doi: 10.1098/rsta.2018.0227 (PMC6562345; doi:10.1098/rsta.2018.0227)
Supplement: Experimental and computational details [file rsta20180227supp4.pdf]

Hydrogen-bond-mediated structural variation of metal guanidinium formate hybrid perovskites under pressure

Z. Yang, G. Cai, C. L. Bull, M. G. Tucker, M. T. Dove, A. Friedrich, and A. E. Phillips

Phil. Trans. Royal Soc. A
DOI:10.1098/rsta.2018.0227

Electronic Supplementary Information

Contents

1 Single-crystal X-ray diffraction **S1**

2 Powder neutron diffraction **S7**

3 Density-functional theory **S14**

1 Single-crystal X-ray diffraction

Single-crystal laboratory X-ray diffraction data were collected in house at Goethe University Frankfurt am Main, using an XCalibur3 four-circle diffractometer from Oxford Diffraction equipped with a Sapphire3 CCD camera and a sealed tube with Mo $K\alpha$ radiation. The sample-to-detector distance was 73 mm, the scan width 0.5°, and the exposure time 60 s. At this pressure the crystal was already in the high-pressure phase. The phase transition was observed by the formation of zoning, *i.e.*, of parallel twin domains in the crystal (Fig. S1). Twin data reduction of two components and empirical absorption correction were performed with the CrysAlisPro software (version 1.171.37.31).[1] The crystal structure was solved in space group $R\bar{3}c$ using direct methods (SHELXS) and Fourier expansion technique.[2] All non-hydrogen atoms were refined in anisotropic approximation, with hydrogen atoms 'riding' in idealised positions, by full-matrix least squares against F^2 of all data, using SHELXL software.[2] The crystal was a non-merohedral twin with domains rotated by 179.6° around real axis $[0.82\ 0.41\ 0.41]$ or reciprocal axis $(0.35\ 0.00\ 0.94)$. Reflections from both domains were used in the refinements. The twin component was refined to about 37%.

Single-crystal synchrotron diffraction data were collected on beamline I19 at Diamond Light Source, UK, as described in the main text. The raw data are archived in the Diamond Light Source archive as experiment number MT17851.

Initially all data sets were reduced automatically using `xia2`:[3] the raw images were integrated with `DIALS`;[4] an empirical absorption correction was applied and the data scaled using `ccp4`,[5] `pointless`,[6] and `aimless`.[7] In a few cases where this automatic process failed, data were reduced by hand using CrysAlisPro (version 1.171.38.46).[1] This was especially the case for the high-pressure, twinned data sets, where the two data reduction programmes were complementary. `xia2` has a well-established instrument

model for data from the I19 beamline, but it is not set up to deal with non-merohedral twinning. Since the two twin components overlap in only a single plane of reciprocal space, it was possible in some cases to integrate peaks from a single twin domain, but this is clearly not ideal. On the other hand, CrysAlisPro is very sensitive to the precise instrument model used; although this was refined against a good-quality test data set collected immediately before the experiment reported here, the data reduced using this method are less internally consistent (have higher R_{int}) than those reduced using `xia2`. However, this program is easily capable of dealing with non-merohedral twinning, so that subsequent structure refinement can incorporate information from both domains. Incorporating both twin domains has the further advantage of enabling twice as many diffraction peaks to be used in the refinement, which is important since a significant proportion were blocked by the diamond anvil cell.

For this reason, in one case below we give refinements of data integrated in both ways from the same raw images. The differences between these refinements are clearly artefacts of the software and approach used rather than the raw data themselves. In particular, using `xia2` the data were only integrated up to 0.8 Å, while using CrysAlisPro all predicted spots were integrated but an appropriate resolution cutoff was applied subsequently; thus the total number of reflections is not directly comparable between data sets.

The twin law found from indexing the synchrotron data was a rotation of $\phi = 111.0^\circ$ about the reciprocal axis (101) (Fig. S2). This is equivalent within experimental error to the description found from the laboratory data. As noted in the main text, this specific twin law is not a coincidence; it corresponds to an angle θ between the planes of the guanidinium atoms given from the laboratory twin law by

$$\theta = \cos^{-1}\left(\frac{-3a^2+c^2}{3a^2+c^2}\right) = 97.8^\circ$$

or from the synchrotron twin law by

$$\theta = \cos^{-1}\left(\frac{3a^2+4c^2\cos\phi}{3a^2+4c^2}\right) = 98.1^\circ.$$

This is in good agreement with the angle between these planes in the low-pressure "herringbone" pattern, $\theta = 97.7^\circ$ at 0.96 GPa.

For the synchrotron data, the crystal structures were solved using dual-space methods (SHELXT), which found all non-hydrogen atoms immediately without reference to a Fourier difference map.[8] Again, all non-hydrogen atoms were refined in anisotropic approximation, with hydrogen atoms 'riding' in idealised positions, by full-matrix least squares against F^2 of all data, using SHELXL.[2] Rigid bond restraints (RIGU) were applied to constrain the relative motion of all pairs of bonded atoms to be perpendicular to the bond.[9] In a few cases, a spherical restraint was necessary to prevent one atom's displacement parameters from becoming non-positive definite, due to the relatively small number of reflections that could be observed using the diamond anvil cell.

Summary tables of these data are provided below as Tables S1, S2 and S3; full details of the single-crystal data sets collected are available as Crystallographic Information Files (CCDC deposition numbers 1860767–1860777).

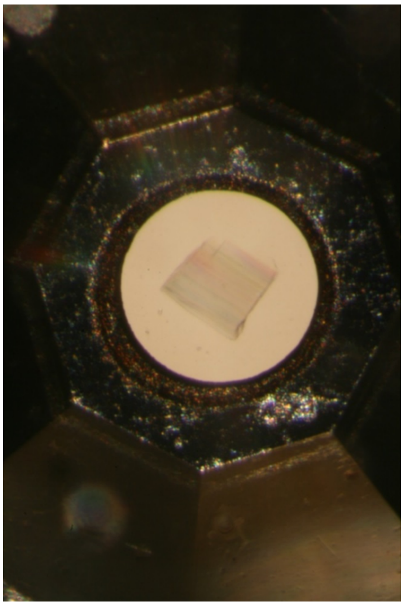

Figure S1: Single crystal of MnGF at a pressure of $1.70(3)$ GPa in the high-pressure phase. Parallel twin domains are observed in the crystal, formed at the high-pressure phase transition. The diameter of the culet is approximately $600\,\mu m$, the diameter of the hole is approximately $400\,\mu m$, and the diameter of the crystal is approximately $250\,\mu m$.

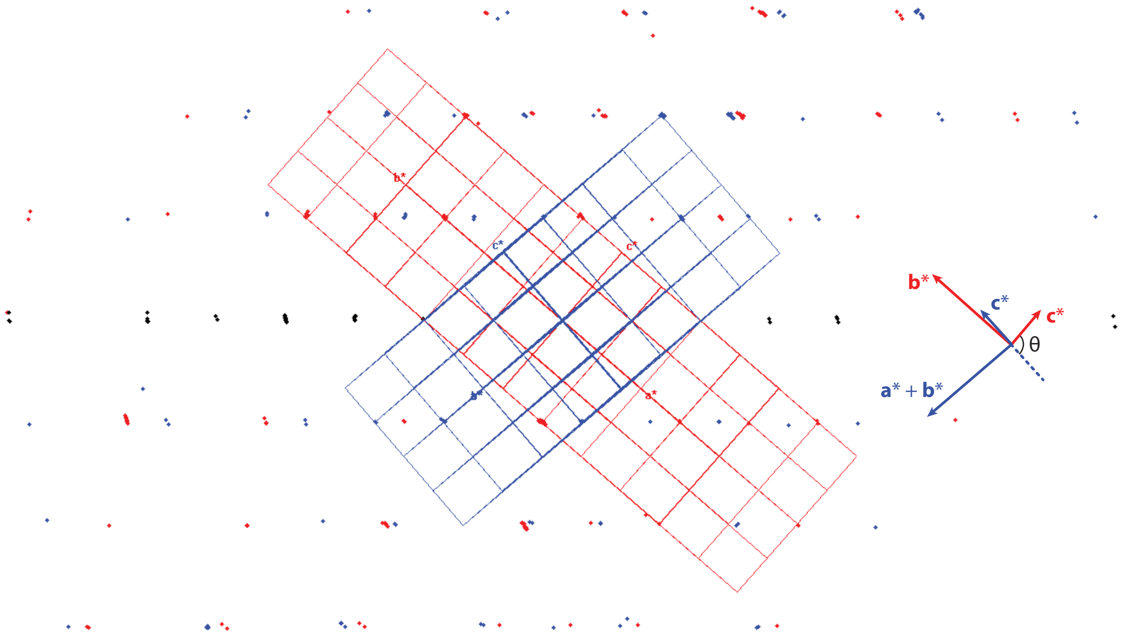

Figure S2: Strong reflections observed during the synchrotron experiments on the high-pressure phase of **MnGF**, coloured red or blue according to their origin from the two twin components. Overlapping reflections are shown in black.

Table S1: Summary of the single-crystal structure refinement against laboratory data for **MnGF**.

Chemical formula and mass ($g\,mol^{-1}$)	$C_4H_9MnN_3O_6$, 250.08
T (K)	298(2)
Wavelength (Å)	0.71073, Mo $K\alpha$
p (GPa)	1.70(3)
Crystal system and space group	Trigonal, $R\bar{3}c$ (no. 167)
Z	6
Reduction software	CrysAlisPro
a (Å)	8.9825(5)
c (Å)	18.175(3)
V ($Å^3$)	1270.0(3)
ρ ($g\,cm^{-3}$)	1.962
Absorption coefficient μ (mm^{-1})	1.571
θ_{min} (°)	4.513
θ_{max} (°)	26.624
Reflections collected	8400
Unique reflections	619
Unique reflections ($I > 2\sigma(I)$)	505
R_{int}	0.0960
Parameters	25
Restraints	0
Twin fraction	0.37
Goodness-of-fit on F^2	1.111
$R1(I > 2\sigma(I))$	0.0685
$wR2(I > 2\sigma(I))$	0.2068
$R1$ (all data)	0.0811
$wR2$ (all data)	0.2166
Largest peak ($e\,Å^{-3}$)	0.719
Deepest hole ($e\,Å^{-3}$)	−0.506

Table S2: Summary of the single-crystal structure refinements against synchrotron data for **MnGF**.

Chemical formula and mass (g mol^{-1})	$C_4H_9MnN_3O_6$, 250.08					
T (K)	293(2)					
Wavelength (Å)	0.4859					
p (GPa)	0	0.63	0.96	1.70	2.14	
Crystal system and space group	Orthorhombic, *Pnna* (no. 52)			Trigonal, $R\bar{3}c$ (no. 167)		
Z	4			6		
Reduction software	xia2	xia2	xia2	CrysAlisPro	CrysAlisPro	xia2
a (Å)	8.5145(11)	8.3814(13)	8.2781(9)	9.041(2)	9.030(3)	8.938(10)
b (Å)	11.9867(11)	11.8640(13)	11.7712(8)			
c (Å)	9.0577(9)	9.0589(11)	9.0479(7)	18.091(5)	17.969(7)	17.63(4)
V (Å^3)	924.44(17)	900.8(2)	881.66(13)	1280.5(7)	1268.9(10)	1220(4)
ρ (g cm^{-3})	1.797	1.844	1.884	1.946	1.964	2.043
Absorption coefficient (mm^{-1})	0.498	0.511	0.522	0.539	0.544	0.566
θ_{min} (°)	2.244	1.934	1.941	2.352	2.360	2.394
θ_{max} (°)	20.009	19.969	19.967	16.394	16.376	16.551
Reflections collected	3081	3257	1805	4342	4276	449
Unique reflections	821	809	684	452	432	147
Unique reflections ($I > 2\sigma(I)$)	470	507	391	305	283	88
R_{int}	0.0748	0.0867	0.0981	0.2312	0.2143	0.0978
Parameters	66	66	66	25	25	24
Restraints	36	36	36	12	12	12
Twin fraction				0.443(11)	0.441(11)	
Goodness-of-fit on F^2	0.609	0.875	1.017	1.127	1.041	0.779
$R1(I > 2\sigma(I))$	0.0322	0.0388	0.0612	0.1041	0.1063	0.0766
$wR2(I > 2\sigma(I))$	0.0812	0.0851	0.1508	0.2585	0.2544	0.2023
$R1$ (all data)	0.0664	0.0761	0.2001	0.1370	0.1343	0.1071
$wR2$ (all data)	0.1002	0.1033	0.1760	0.2951	0.2762	0.2236
Largest peak (e Å^{-3})	0.254	0.315	0.636	1.189	1.086	0.726
Deepest hole (e Å^{-3})	−0.272	−0.338	−1.009	−1.198	−1.278	−0.681

Table S3: Summary of the single-crystal structure refinements against synchrotron data for **CoGF**.

Chemical formula and mass ($g\,mol^{-1}$)	$C_4H_9CoN_3O_6$, 254.07			
T (K)	293(2)			
Wavelength (Å)	0.4859			
p (GPa)	0	0.88	1.21	1.75
Crystal system and space group	Orthorhombic, *Pnna* (no. 52)			
Z	4			
Reduction software	xia2	CrysAlisPro	xia2	xia2
a (Å)	8.319(8)	8.229(4)	8.141(12)	8.020(2)
b (Å)	11.735(5)	11.6518(18)	11.583(5)	11.4685(15)
c (Å)	8.920(4)	8.913(3)	8.896(8)	8.908(3)
V ($Å^3$)	870.8(10)	854.0(5)	838.9(15)	819.3(4)
ρ ($g\,cm^{-3}$)	1.938	1.976	2.012	2.060
Absorption coefficient (mm^{-1})	0.687	0.701	0.714	0.731
θ_{min} (°)	2.289	1.966	2.318	2.428
θ_{max} (°)	17.695	17.668	18.637	17.643
Reflections collected	1511	2952	1445	1533
Unique reflections	399	613	433	384
Unique reflections ($I > 2\sigma(I)$)	201	315	208	236
R_{int}	0.0759	0.1570	0.0751	0.1158
Parameters	66	66	66	66
Restraints	42	36	42	36
Goodness-of-fit on F^2	0.875	1.066	0.931	1.003
$R1(I > 2\sigma(I))$	0.0387	0.0868	0.0499	0.0705
$wR2(I > 2\sigma(I))$	0.0802	0.1980	0.0814	0.1290
$R1$ (all data)	0.0866	0.1691	0.1188	0.1142
$wR2$ (all data)	0.0935	0.2476	0.0973	0.1477
Largest peak ($e\,Å^{-3}$)	0.523	0.789	0.417	0.467
Deepest hole ($e\,Å^{-3}$)	−0.396	−0.806	−0.461	−0.409

2 Powder neutron diffraction

Powder samples were finely ground and loaded into a zirconia-toughened alumina (ZTA) anvil within the ISIS Paris-Edinburgh press; data were collected on PEARL as described in the main text. The raw data are stored in the ISIS archive, available at `https://data.isis.stfc.ac.uk`, under the run numbers tabulated below, which also indicate the order in which data sets were collected.

The data were processed using Mantid.[10] Diffractometer constants were calibrated against NIST silicon standards. Rietveld refinements were performed starting from models from single-crystal diffraction using TOPAS-Academic 5.[11] The refinements included four phases: the material under study, lead (as pressure sensor), zirconia, and alumina (from the anvils). The pressure was determined from the refined lattice parameter of lead, referred to the third-order Birch-Murnaghan equation of state.

Observed and modelled powder diffraction patterns at various pressures are shown in Figure S3. Full representative refinements in each phase of each material are shown in Figures S4 and S5. Cell parameters are tabulated in Tables S4, S5, S6, and S7 below.

The experimental crystallographic unit cell volumes (Fig. 2c,d) were fit to the second-order Birch-Murnaghan equation of state [12, p 70]:

$$P = \tfrac{3}{2}B_0\left[\left(\frac{V_0}{V}\right)^{\frac{7}{3}} - \left(\frac{V_0}{V}\right)^{\frac{5}{3}}\right] \qquad (1)$$

This model has the advantage of accounting for finite strain and changes in bulk modulus with pressure without including the derivative $B' = \partial B/\partial P$ as a separate parameter: our data do not cover a sufficient range to fit B' precisely. It was also used to fit the DFT data; see §3 below. The fitted parameters are tabulated in Table S8 below.

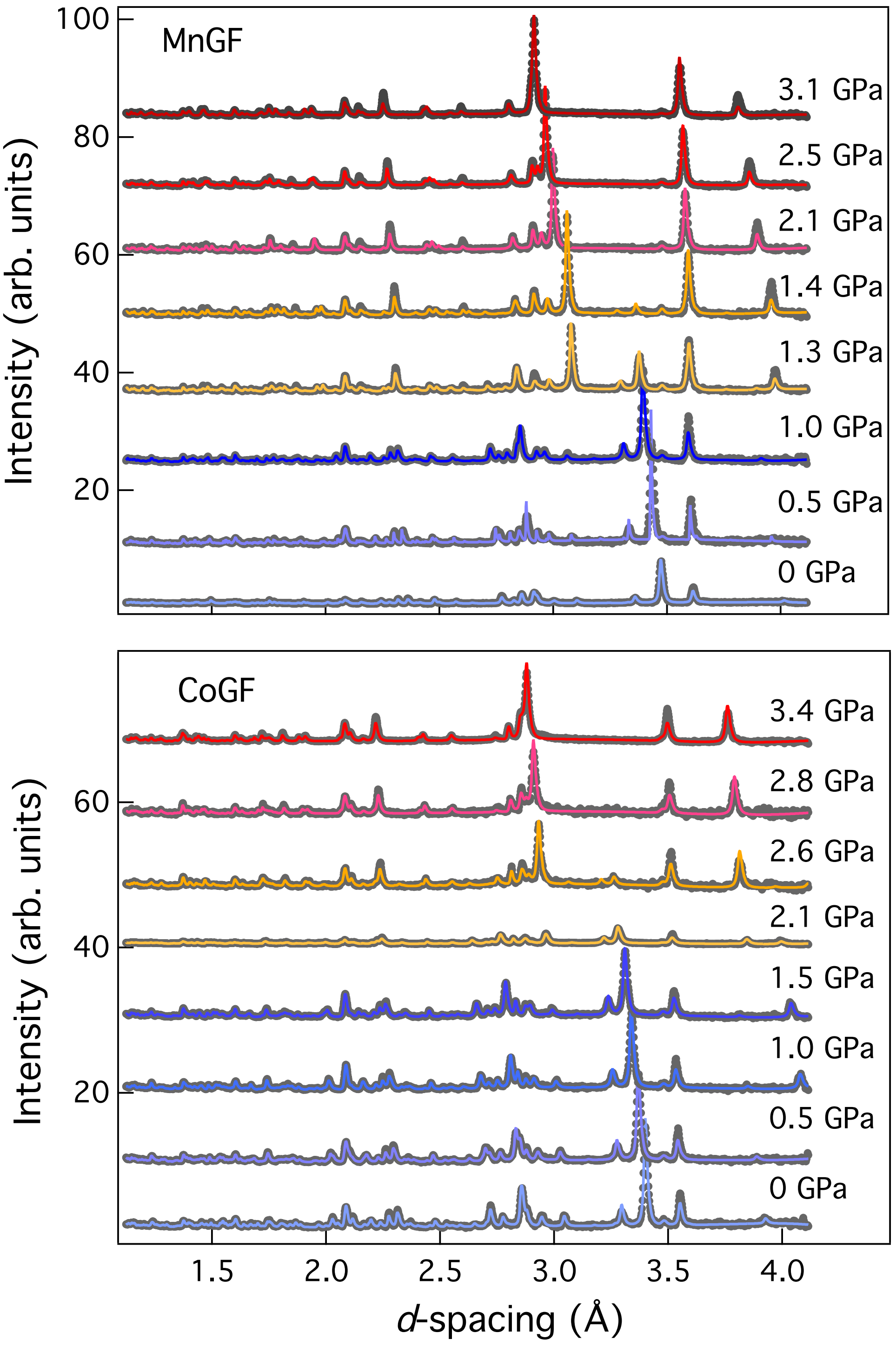

Figure S3: Observed and modelled powder diffraction patterns at different pressures for **MnGF** (top) and **CoGF** (bottom). In each case blue represents the orthorhombic phase; red the rhombohedral phase; yellow the region where both coexist.

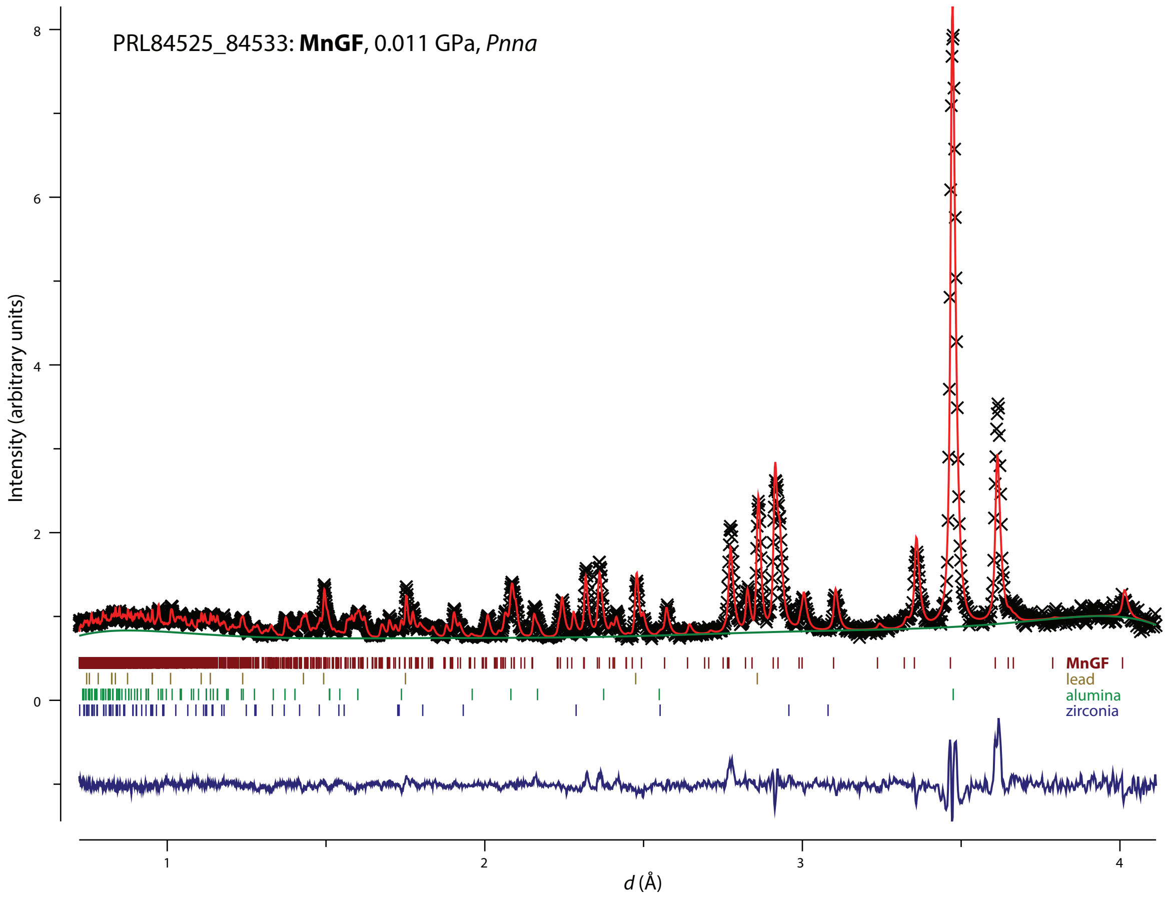

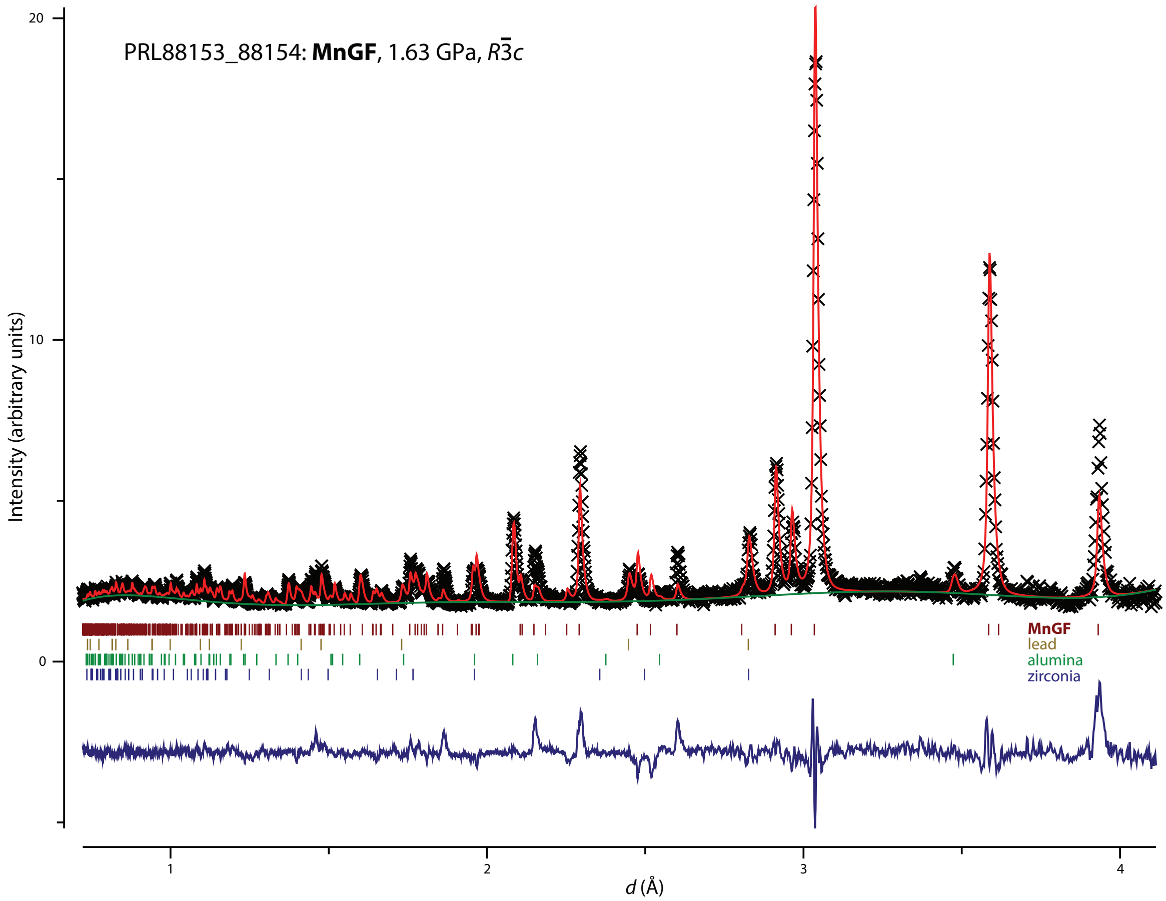

Figure S4: Sample Rietveld refinements for **MnGF** in the low- (top) and high-pressure (bottom) phases.

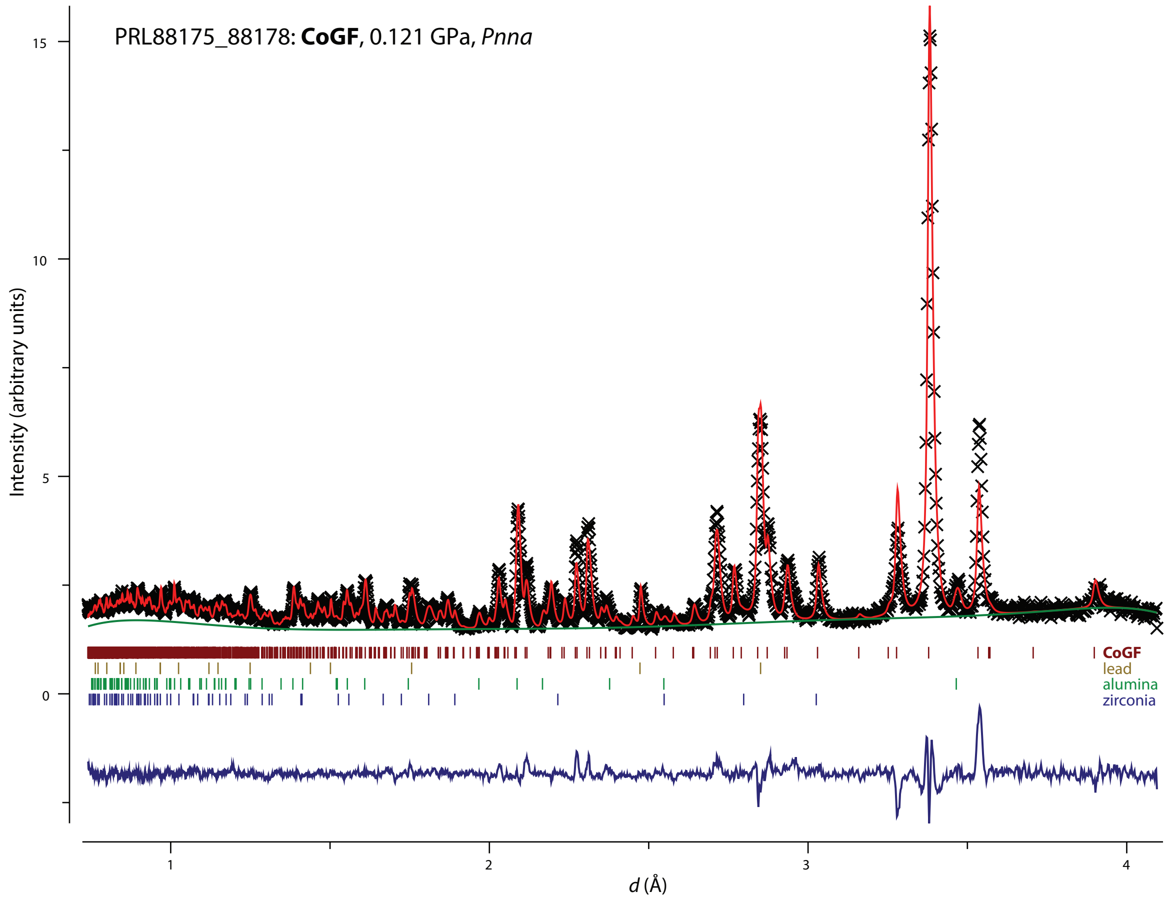

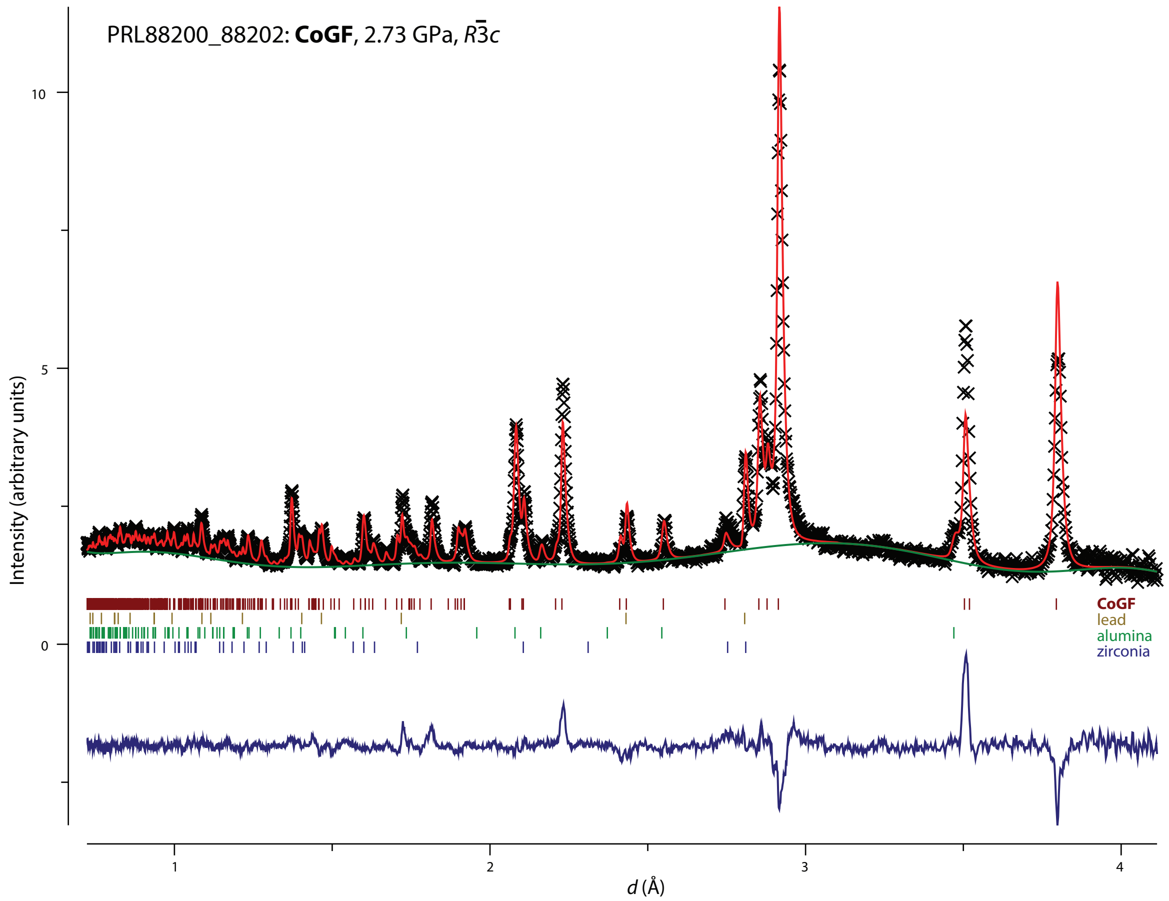

Figure S5: Sample Rietveld refinements for **CoGF** in the low- (top) and high-pressure (bottom) phases.

Table S4: Refined cell parameters, from powder diffraction data, as a function of pressure for **MnGF** in the *Pnna* phase.

Run no.	R_{wp}	p (GPa)	a (Å)	b (Å)	c (Å)	V (Å^3)
PRL84525–84533	3.83	0.012(5)	8.5146(4)	11.9709(8)	9.0539(7)	922.84(10)
PRL87536–87537	3.90	0.047(7)	8.5118(5)	11.9659(9)	9.0480(7)	921.54(11)
PRL88134–88135	4.01	0.138(10)	8.4948(4)	11.9522(8)	9.0554(6)	919.41(10)
PRL88136	4.50	0.220(12)	8.4696(5)	11.9337(9)	9.0554(8)	915.26(12)
PRL88137–88138	4.13	0.403(11)	8.4244(5)	11.8939(9)	9.0544(7)	907.18(11)
PRL88139	4.37	0.513(12)	8.3993(5)	11.8749(9)	9.0543(8)	902.35(8)
PRL88140–88141	3.92	0.665(12)	8.3687(5)	11.8510(8)	9.0530(7)	897.82(8)
PRL84534–84541	3.74	0.677(6)	8.3557(5)	11.8418(8)	9.0509(7)	895.55(11)
PRL88142	4.35	0.713(13)	8.3540(5)	11.8398(9)	9.0537(8)	894.92(8)
PRL88143	4.42	0.796(26)	8.3409(3)	11.8289(6)	9.0544(5)	893.27(11)
PRL88144–88145	3.79	0.966(12)	8.3013(4)	11.7949(8)	9.0525(7)	886.35(10)
PRL88146	4.42	1.017(13)	8.2896(5)	11.7876(9)	9.0519(8)	884.51(11)
PRL88147–88148	3.77	1.112(11)	8.2682(4)	11.7737(8)	9.0526(7)	881.25(10)
PRL88149	4.60	1.183(15)	8.2535(6)	11.7608(10)	9.0534(9)	878.79(12)
PRL88150–88151	4.71	1.250(17)	8.2445(10)	11.740(2)	9.049(2)	875.6(3)
PRL88152	6.95	1.35(3)	8.227(3)	11.648(3)	9.064(5)	868.8(6)

Table S5: Refined cell parameters, from powder diffraction data, as a function of pressure for **MnGF** in the $R\bar{3}c$ phase.

Run no.	R_{wp}	p (GPa)	a (Å)	c (Å)	V (Å^3)
PRL88150–88151	4.71	1.250(17)	9.0101(6)	18.4472(8)	1296.8(2)
PRL88152	6.95	1.35(3)	9.0091(7)	18.3422(9)	1289.3(2)
PRL88153–88154	6.73	1.64(2)	9.0066(5)	18.2053(8)	1278.93(16)
PRL87538	5.91	1.767(12)	8.9985(5)	18.1299(8)	1271.50(11)
PRL88155	6.54	1.82(2)	9.0052(5)	18.1213(8)	1272.53(16)
PRL84542–84549	6.16	1.865(12)	9.0007(5)	18.0560(8)	1266.82(15)
PRL88156–88157	6.45	1.96(3)	9.0025(5)	18.0346(8)	1265.79(15)
PRL88158–88159	6.62	2.06(2)	9.0016(5)	17.9701(8)	1260.99(15)
PRL88160	6.81	2.26(3)	8.9982(5)	17.8879(8)	1254.31(16)
PRL87539–87542	5.76	2.309(12)	8.9903(5)	17.8468(7)	1249.37(15)
PRL88161–88162	6.36	2.50(3)	8.9924(5)	17.7664(8)	1244.16(15)
PRL88163–88164	6.29	2.70(3)	8.9873(5)	17.6732(7)	1236.30(15)
PRL84550–84555	6.17	2.763(13)	8.9829(5)	17.6120(8)	1230.82(10)
PRL87543–87546	5.62	2.795(12)	8.9793(5)	17.6189(8)	1230.26(15)
PRL88165–88166	6.14	2.94(3)	8.9826(5)	17.5736(8)	1227.94(15)
PRL88167–88168	6.25	3.14(3)	8.9766(6)	17.4732(9)	1219.28(16)

Table S6: Refined cell parameters, from powder diffraction data, as a function of pressure for **CoGF** in the *Pnna* phase. Reported negative pressures are artefacts at very low pressure loading of the pressure calibration used, and should not be taken literally.

Run no.	R_{wp}	p (GPa)	a (Å)	b (Å)	c (Å)	V (Å^3)
PRL88173–88174	4.31	−0.011(13)	8.3237(4)	11.7462(8)	8.9055(7)	870.71(10)
PRL88172	5.35	−0.001(15)	8.3246(6)	11.7458(10)	8.9064(8)	870.86(13)
PRL88175–88178	3.78	0.100(9)	8.3063(4)	11.7296(7)	8.9035(6)	867.46(9)
PRL88179–88180	3.93	0.525(11)	8.2380(4)	11.6719(8)	8.8983(7)	855.59(10)
PRL85565–85567	4.42	0.777(12)	8.1838(5)	11.6293(9)	8.8956(8)	846.61(12)
PRL88181	4.55	0.828(13)	8.1797(6)	11.6258(10)	8.8946(8)	845.84(12)
PRL88182–88183	3.65	1.022(10)	8.1497(4)	11.6005(8)	8.8943(7)	840.87(10)
PRL88185–88186	3.75	1.193(10)	8.1201(4)	11.5741(8)	8.8936(7)	835.84(10)
PRL88187–88189	3.42	1.387(9)	8.0919(4)	11.5515(7)	8.8919(7)	831.16(9)
PRL88190–88191	3.64	1.533(10)	8.0689(4)	11.5336(8)	8.8910(7)	827.43(10)
PRL88192–88194	3.44	1.670(10)	8.0487(4)	11.5168(7)	8.8904(7)	824.09(9)
PRL88195–88196	3.60	1.897(11)	8.0201(4)	11.4944(8)	8.8885(7)	819.40(10)
PRL85568–85576	3.55	2.092(11)	7.9824(6)	11.4682(11)	8.8809(11)	812.98(14)
PRL88197–88198	3.99	2.405(13)	7.9392(15)	11.443(3)	8.878(3)	806.4(3)
PRL88199	5.79	2.565(18)	7.932(3)	11.428(5)	8.876(5)	804.5(6)

Table S7: Refined cell parameters, from powder diffraction data, as a function of pressure for **CoGF** in the $R\bar{3}c$ phase.

Run no.	R_{wp}	p (GPa)	a (Å)	c (Å)	V (Å^3)
PRL85568–85576	3.55	2.092(11)	8.8451(16)	17.7537(13)	1202.9(5)
PRL88197–88198	3.99	2.405(13)	8.8398(6)	17.6308(7)	1193.12(16)
PRL88199	5.79	2.565(18)	8.8366(8)	17.5676(9)	1188.0(2)
PRL88200–88202	4.61	2.731(15)	8.8310(5)	17.4828(7)	1180.76(14)
PRL88203	6.88	2.809(24)	8.8306(8)	17.4310(10)	1177.2(2)
PRL88204–88208	4.55	3.205(15)	8.8236(5)	17.3165(7)	1167.62(14)
PRL88209–88212	4.60	3.382(15)	8.8199(5)	17.2526(7)	1162.28(14)
PRL85577–85579	5.51	3.441(18)	8.8161(6)	17.2248(8)	1159.41(15)

Table S8: Parameters of the second-order Birch-Murnaghan equation of state fitted to powder neutron diffraction data. Volumes are given per formula unit. Uncertainties are estimated from the fitting procedure only.

Material	Phase	V_0 (Å^3)	B_0 (GPa)
**MnGF**	*Pnna*	231.03(9)	21.3(3)
	$R\bar{3}c$	227.1(4)	22.6(5)
**CoGF**	*Pnna*	217.65(9)	27.2(3)
	$R\bar{3}c$	214.9(6)	26.2(10)

3 Density-functional theory

DFT calculations were performed using CASTEP 18.1,[13] which uses the plane-wave pseudopotential method.[14] The PBEsol functional[15] was combined with the Tkatchenko-Scheffler semi-empirical dispersion correction.[16] The basis set contained plane waves up to an energy of 650 eV; the fine grid used for augmentation charges was five times as dense as the basis grid. The tolerance for self-consistent field convergence was 10^{-9} eV atom^{-1}. Calculations on **MnGF** and **CoGF** were spin-polarised and started from a G-type (3D checkerboard) antiferromagnetic configuration.

For each phase the geometry (*i.e.*, unit cell parameters and atomic positions) was initially optimised freely within the given space-group symmetry. Then copies of the optimised structure were created in which the relative atomic coordinates remained the same while the total cell volume changed in steps of approximately 1%. Each of these configurations was allowed to relax again while maintaining constant volume. The criteria for geometry convergence were a change in energy per ion per iteration of 2×10^{-5} eV, a maximum force of 5×10^{-2} eV Å^{-1}, a maximum ionic translation of 1×10^{-3} Å, and, nominally, a maximum stress component of 0.1 GPa. For cell volumes far from equilibrium, this stress criterion was waived where necessary: that is, in cases where the maximum stress component had clearly converged with respect to the cell geometry.

The energies calculated in this way were fitted as a function of volume to the second-order Birch-Murnaghan equation of state:

$$E(V) = E_0 + \tfrac{9}{8} B_0 \left[V_0 + V \left\{ \left(\frac{V}{V_0} \right)^{\frac{7}{3}} - 2 \left(\frac{V}{V_0} \right)^{\frac{5}{3}} \right\} \right]. \qquad (2)$$

This corresponds to the same model as equation (1) above, and has only three parameters: the equilibrium energy E_0, volume V_0, and bulk modulus B_0. The fitted parameters are tabulated below as Table S9.

Table S9: Parameters of the second-order Birch-Murnaghan equation of state fitted to DFT data. Energy and volume are both given per formula unit, and energy is relative to the minimum of the rhombohedral phase. Uncertainties are estimated from the fitting procedure only.

Material	Phase	E_0 (meV)	V_0 (Å^3)	B_0 (GPa)
**MnGF**	*Pnna*	−38.0(12)	226.95(7)	37.6(10)
	$R\bar{3}c$	0	222.0(3)	25.3(10)
**CoGF**	*Pnna*	−22.4(12)	213.13(6)	36.5(8)
	$R\bar{3}c$	0	208.0(2)	30.2(12)
**CdGF**	*Pnna*	37.6(12)	244.96(13)	26.3(11)
	$R\bar{3}c$	0	240.4(3)	15.7(8)

References

(1) Rigaku Oxford Diffraction, Oxford, U.K. CrysAlisPRO Software System., 2015.

(2) Sheldrick, G. M. *Acta Crystallogr. A* **2008**, *64*, 112–122.

(3) Winter, G. *J. Appl. Crystallogr.* **2010**, *43*, 186–190.

(4) Parkhurst, J. M.; Winter, G.; Waterman, D. G.; Fuentes-Montero, L.; Gildea, R. J.; Murshudov, G. N.; Evans, G. *J. Appl. Crystallogr.* **2016**, *49*, 1912–1921.

(5) Winn, M. D. et al. *Acta Crystallogr. D* **2011**, *67*, 235–242.

(6) Evans, P. *Acta Crystallogr. D* **2006**, *62*, 72–82.

(7) Evans, P. R.; Murshudov, G. N. *Acta Crystallogr. D* **2013**, *69*, 1204–1214.

(8) Sheldrick, G. M. *Acta Crystallogr. A* **2015**, *71*, 3–8.

(9) Thorn, A.; Dittrich, B.; Sheldrick, G. M. *Acta Crystallogr. A* **2012**, *68*, 448–451.

(10) Arnold, O. et al. *Nucl. Instrum. Meth. A* **2014**, *764*, 156–166.

(11) Coelho, A. A. *J. Appl. Crystallogr.* **2018**, *51*, 210–218.

(12) Poirier, J.-P., *Introduction to the Physics of the Earth's Interior*, second edition; Cambridge University Press: Cambridge, 2004.

(13) Clark, S. J.; Segall, M. D.; Pickard, C. J.; Hasnip, P. J.; Probert, M. J.; Refson, K.; Payne, M. *Z. Kristall.* **2005**, *220*, 567–570.

(14) Payne, M. C.; Teter, M. P.; Allan, D. C.; Arias, T.; Joannopoulos, J. D. *Rev. Mod. Phys.* **1992**, *64*, 1045–1097.

(15) Perdew, J. P.; Ruzsinszky, A.; Csonka, G. I.; Vydrov, O. A.; Scuseria, G. E.; Constantin, L. A.; Zhou, X.; Burke, K. *Phys. Rev. Lett.* **2008**, *100*, 136406.

(16) Tkatchenko, A.; Scheffler, M. *Phys. Rev. Lett.* **2009**, *102*, 073005.
